# Correction: A new family of fullerene derivatives: fullerene–curcumin conjugates for biological and photovoltaic applications

**DOI:** 10.1039/c9ra90002k

**Published:** 2019-01-18

**Authors:** Edison Castro, Maira R. Cerón, Andrea Hernandez Garcia, Quentin Kim, Alvaro Etcheverry-Berríos, Mauricio J. Morel, Raúl Díaz-Torres, Wenjie Qian, Zachary Martinez, Lois Mendez, Frank Perez, Christy A. Santoyo, Raquel Gimeno-Muñoz, Ronda Esper, Denisse A. Gutierrez, Armando Varela-Ramirez, Renato J. Aguilera, Manuel Llano, Monica Soler, Núria Aliaga-Alcalde, Luis Echegoyen

**Affiliations:** Department of Chemistry, The University of Texas at El Paso 500W University Ave El Paso TX 79968 USA eacastroportillo@utep.edu; Physical and Life Sciences, Lawrence Livermore National Laboratory 7000 East Ave Livermore CA 94550 USA; Departamento de Ingeniería Química, Biotecnología y Materiales, Facultad de Ciencias Físicas y Matemáticas, Universidad de Chile Beauchef 851 Santiago Chile; CSIC-ICMAB (Institut de Ciéncia dels Materials de Barcelona), Campus de la Universitat Autónoma de Barcelona 08193 Bellaterra Spain; Department of Biological Sciences, Border Biomedical Research Center, The University of Texas at El Paso 500 West University Avenue El Paso TX 79968 USA; ICREA (Institució Catalana de Recerca i Estudis Avançats) Passeig Lluís Companys 23 08010 Barcelona Spain

## Abstract

Correction for ‘A new family of fullerene derivatives: fullerene–curcumin conjugates for biological and photovoltaic applications’ by Edison Castro *et al., RSC Adv.*, 2018, **8**, 41692–41698.

The authors regret that the following funding information was mistakenly omitted from the acknowledgements in the original article: “Research reported in this article was partially supported by the National Institute of General Medical Sciences of the National Institutes of Health under linked Award Numbers RL5GM118969, TL4GM118971, and UL1GM118970. The content is solely the responsibility of the authors and does not necessarily represent the official views of the National Institutes of Health.”

The Royal Society of Chemistry apologises for these errors and any consequent inconvenience to authors and readers.

## Supplementary Material

